# Development of a genetic linkage map of rubber tree (*Hevea braziliensis*) based on microsatellite markers

**DOI:** 10.1186/1753-6561-5-S7-P39

**Published:** 2011-09-13

**Authors:** Livia Moura Souza, Camila Campos Mantello, Fernando Suzuki, Rodrigo Gazaffi, Dominique Garcia, Vincent Le Guen, Antonio Augusto Franco Garcia, Anete Pereira Souza

**Affiliations:** 1 Molecular Biology Center and Genetic Engeniering, UNICAMP, Campinas, SP,13083-875, Brazil; 2 Department of Genetics "Luiz de Queiroz" College of Agriculture University of São Paulo, Piracicaba, SP,13400-970, Brazil; 3 CIRAD-BIOS, UMR AGAP, Montpellier, Cedex 5, France; 4 Department of Plant Biology – Biology Institute and Molecular Biology Center and Genetic Engeniering, UNICAMP, Campinas, SP,13083-875, Brazil

## Background

Rubber tree (*Hevea* spp.) is the main natural rubber producing crop, and it is cultivated in numerous equatorial, tropical and subtropical areas. *Hevea* spp. is an outcrossing perennial species and belongs to the botanical family Euphorbiaceae. Breeding of rubber tree, like for many other tree crops, is a long term process. Large scale cultivation of a new cultivar can only be reached after 20–25 years of field experiments on large areas. Breeders are thus interested in developing alternative strategies to improve and speed up the breeding scheme, using genetic markers. Molecular markers are being used for more than 15 years in *Hevea* breeding, mostly for diversity studies, assessment of genetic distance among cultivars, genetic mapping and identification of genetic loci implied in the expression of agronomical traits.

## Material and methods

The mapping population was a full-sib progeny (F1 progeny) derived from a controlled cross between the cultivars PB217 and PR255. The PB217 has a high yield potential, which is expressed throughout the lifetime of its rubber production, low metabolic activity and a high level of sucrose in the latex vessels. Genotype PR255 showed tolerance to injury and cold. A set of 603 microsatellite primer pairs was tested for polymorphism the two parents and six F1 progenies genotypes. Among these, 200 genomic microsatellites have been developed by our laboratory (part published in [7]), 296 were developed by CIRAD [3] and 178 EST-SSRs were obtained from a published work [1]. The polymorphic microsatellites between the parents were selected for mapping in a F1 population. The PCR conditions were detailed by [7]. PCR products were separated using a DNA analyzer 4300 (LI-COR) through 6.5% polyacrylamide gels. Linkage map was obtained using OneMap [5]. This software generates integrated genetic maps from molecular markers with different segregation patterns; it considers multipoint technology based on Hidden Markov Models, as presented by [8]. LOD Score 4.5 and recombination fraction of 0.40 was considered to determine linkage between markers. The algorithms “compare" (until six markers) and "order" (more than six) were applied to obtain best order for each linkage groups, as presented in mapmaker/EXP [6].

## Results and discussion

Out of 603 microsatellites markers evaluated, 309 (51%) showed polymorphism between the parents of the mapping population. Until now, 225 marks were genotyped (59 SSR genomic loci and 166 EST-SSR). Chi-square test was carried out on the genotyping polymorphic loci showed that 110 loci followed a segregation ratio of 1:1, 28 followed a ratio of 1:2:1 and 87 (38.7%) followed a ratio of 1:1:1:1. The map consists of 225 markers, distributed in 23 linkage groups (LG) and 2,471.2 cM in length with an average genetic distance of 11 cM between adjacent markers. The largest group has 215.9 cM (18 markers) and the smallest has 2.71 cM (2 markers). This reflects in a more realistic way the polymorphism of the full-sib cross.

In a previous work [4] a linkage map was constructed from a double pseudo testcross, and two maps were constructed separately for each parent. In the present work, instead of using only single dose markers (1:1) like [4], markers that segregate in both parents in the ratio 1:2:1 and 1:1:1:1 were also used enabling the construction of an integrated genetic map and facilitating the location of quantitative trait loci (QTL) [2]. Seven linkage groups are small, composed of two or three markers and covering a few cM. These small linkage groups probably occur because the map is not saturated enough and some chromosome regions could not be linked. The chromosome number accepted today, for most *Hevea* species, is 18 (2n = 36), as observed by [4].

## Conclusions

This map is still under development and we expect to achieve a saturated map consisting of 18 linkage groups. The present map will be used for yield rubber QTL mapping and other important economical characteristics.
